# On Ruptures of the Uterus

**Published:** 1821-11

**Authors:** Thomas Purton

**Affiliations:** Member of the Royal College of Surgeons.


### *On Ruptures of the Uterus.* By Thomas Purton, Member of the Royal College of Surgeons.

THE perusal of Dr. Ramsbotham's "Practical Observations in Midwifery," &c. has recalled to my mind very many cases of interest, which have occurred to me during an extensive practice, and which I greatly regret were not published at the time, whilst they were fresh in my memory. His observations on ruptures of the uterus induce me to think that I should not feel justified in withholding the following communication relative to that important subject; convinced, as I am, that it is the duty of every accoucheur to publish (let it be favourable or otherwise,) each interesting case occurring within his practice, as, by so doing, he may be often rendering to his medical brethren the most important service.

—— Joins, a poor woman of Kinwarton, an adjoining parish to Alcester, the mother of ten children at nine births, died, undelivered of her last child, from a rupture of the uterus. The six first labours were not attended (as I can recollect,) with any particulars worthy of notice. The seventh labour (May 22d, 1811,) proceeded very well, till the head had entered low in the pelvis; the pains were strong and forcing; when all at once she screamed out, "Do deliver me! I shall die! Oh, the pain, the pain!" This acute pain, so different from that of labour, continued without intermission till she was delivered, which was easily effected with the assistance of the forceps. The placenta soon followed, but with no cessation of pain. In the course of three hours, the abdomen became more distended than before delivery; and, by the next morning, every symptom of peritoneal inflammation had taken place: yet, with all these alarming symptoms, she recovered. It may be worthy of notice, that the lochiæ were trifling; but, *per rectum,* there was a prodigious discharge of a bloody, watery fluid, which commenced early in the evening of the second day, and continued more or less for nearly a fortnight.

May 10th, 1813, she again fell in labour; the first part of which proceeded regularly and well, till the head of the child was on the point of entering the brim of the pelvis: the pains were strong, but no farther progress was made for some hours. Recollecting the issue of the preceding labour, I called in the assistance of my father-in-law, Mr. Bloxam, who deemed it necessary to deliver by the crotchet, which was done with extreme difficulty, from the great size of the child, being more than twelve pounds in weight. Peritoneal inflammation took place here as in the preceding case, and attended nearly with the same set of symptoms; but she got well by a similar mode of treatment.

July 22d, 1814, she was again in labour, which proceeded gradually till the head entered the pelvis; the pains were strong, and I thought every thing was going on well, till the head began to bear on the tuberosity of the ischium; and, as it then did not perceptibly advance, although the action of the uterus continued strong, I was preparing (having the two preceding cases fresh in my mind,) to deliver by the forceps, when she uttered the most horrible scream I ever heard; and, on my making an examination, the head of the child had wholly receded, the os uteri being also perfectly closed. In fact, the child, with the placenta, had passed through a rupture into the cavity of the abdomen. I was not permitted to have recourse to the Cæsarean operation, or to examine the parts after death. The poor sufferer died on the third day from the accident.

*Alcester; August* 14, 1821.

N. B. The medical treatment of the seventh labour was as follows:

*May 22d.*—℞. Pulv. antim. gr. iv.; Hydrarg. submuriat. gr. ij.; Conf. q. s. ft. pil. 4tis horis sumend. cum mist. anod. commun.

*May* 23.—Cap. cochl. j. larg. ol. ricini ex theæ menthæ cyatho 4tis horis donec alv. resp.

℞. Ol. olivar. ℥j.; Aq. menth. piper. ℥ij.; Magnes. sulphat. ℥ss.; ft. mistura in ℔j. theæ anthemid. pro enemate administ.

*24th.*—℞. Hydrargyri submuriat. gr. iij.; ft. pil. cū confectione statim sumenda.

℞. Acet. distillat. ℥jss.; Potassæ subcarb. q. s.; Lact. amygdal. ℥ivss.; (cū aq. menth. in loco aq. distillat.) Tinct. opii, gutt. xx.; M. cap. ℥jss. 4tis horis.

℞. Liquor ammon. acetat. ℥jss.; Liquor ammoniæ, ℥ss.; Liniment. camph. ℥j. M. ft. embrocat. quacum affric. abdomin. region. bis vel ter de die.

*28th.*—℞. Opii crud. gr. ij.; Pulv. antimon. gr. vj.; Conf. q. s. ft. pil. iij. Cap. i. omni nocte horâ somni. Adde mist. salin, (Cetacei, ʒj.; Mucilag. acaciæ, ʒij. Pulv. ipecac. gr. iv.) et in loco aq. menth. pone mist. camphor. Administ. enema, cū opio omni nocte.

*June* 1.—℞. Opii, gr. ij.; Extract. conii, gr. ix.; ft. pil. iij. Cap. i. omni nocte.

*2d.*—℞. Misturæ camphor. ℥vss.; Conf. amygdal. ℥ss.; Cetacei, ʒj.; Mucilag. acaciæ, ʒij.; Tinct. opii, gutt. xx.; Acid. sulph. dilut. gutt. xxv. M. cap. ℥jss. 4tis horis.

*3d.*—Repetantur pil. anod.; enema; embrocat. et mist. cetacei, quotidiè.

*9th.*—℞. Decoct. cinchonæ, ℥v.; Cetacei, ʒj.; Mucilag. acaciæ, ℥ss.; Spir. æther. sulphurici, ʒiij.; Tinct. opii, gutt. xx. M. cap. ℥j. ter quaterve die cū cochl. parv. gutt. seq.

℞.—Acid. sulphur. dilut. ʒjss.; Syr. violæ, ʒvjss. M. Rep. pil. anod. h. s. quotidiè.

*15th.* Rep. mist. cinchon. ℥vj.

*Obs.*—The last mixture with the sulphuric acid was continued to the latter end of June: the other medicaments were omitted from this day, (June 15th.) A peculiar constitutional debility of frame rendered bleeding in any way totally inadmissible.

